# Effects of lobeglitazone on insulin resistance and hepatic steatosis in high-fat diet-fed mice

**DOI:** 10.1371/journal.pone.0200336

**Published:** 2018-07-06

**Authors:** Bong-Hoi Choi, Zhen Jin, Chin-ok Yi, Juhong Oh, Eun Ae Jeong, Jong Youl Lee, Kyung-ah Park, Kyung Eun Kim, Jung Eun Lee, Hyun-Jin Kim, Jong Ryeal Hahm, Gu Seob Roh

**Affiliations:** 1 Department of Nuclear Medicine, College of Medicine, Gyeongsang National University Hospital, Gyeongsang National University, Jinju, Gyeongnam, Republic of Korea; 2 Department of Anatomy and Convergence Medical Science, Bio Anti-aging Medical Research Center, Institute of Health Sciences, College of Medicine, Gyeongsang National University, Jinju, Gyeongnam, Republic of Korea; 3 EZmass Co., Ltd., Jinju, Gyeongnam, Republic of Korea; 4 Department of Thoracic and Cardiovascular Surgery, College of Medicine, Gyeongsang National University Hospital, Gyeongsang National University, Jinju, Gyeongnam, Republic of Korea; 5 Department of Food Science and Technology, Division of Applied Life Sciences (BK21 plus), Institute of Agriculture and Life Science, Gyeongsang National University, Jinju, Gyeongnam, Republic of Korea; 6 Division of Endocrinology and Metabolism, Department of Internal Medicine, Institute of Health Sciences, College of Medicine, Gyeongsang National University, Jinju, Gyeongnam, Republic of Korea; East Tennessee State University, UNITED STATES

## Abstract

Lobeglitazone (Lobe) is a novel thiazolidinedione antidiabetic drug that reduces insulin resistance by activating peroxisome proliferator-activated receptor-gamma (PPARγ). However, the exact mechanisms of antidiabetic effects of Lobe have not been established in an animal model. The aim of this study was to evaluate the hypoglycemic effects of Lobe and investigate possible factors involved in Lobe-enhanced hepatic steatosis in high-fat diet (HFD)-fed mice. Mice were fed an HFD for 15 weeks. Lobe was administrated orally during the last 9 weeks. Lobe treatment significantly reduced insulin resistance and increased expression of hepatic glucose transporter 4 (GLUT4) and PPARs in HFD-fed mice. However, increased body weight and hepatic steatosis were not reduced by Lobe in these mice. Metabolomics fingerprinting showed that several lipogenesis-related hepatic and serum metabolites in HFD-fed mice had positive or negative correlations with Lobe administration. In particular, increased leptin levels during HFD were further increased by Lobe. HFD-induced signaling transducer and activator of transcription 3 (STAT3) phosphorylation in the hypothalamus was increased by Lobe. In addition, immunohistochemical analysis showed more proopiomelanocortin (POMC)-positive neurons in the hypothalamus of HFD-fed mice (with or without Lobe) compared with normal diet-fed mice. Despite improving leptin signaling in the hypothalamus and enhancing insulin sensitivity in HFD-fed mice, Lobe increased body weight and steatosis. Further research is necessary regarding other factors affecting Lobe-enhanced hepatic steatosis and hyperphagia.

## Introduction

Patients with obesity often have insulin resistance, hepatic steatosis, dyslipidemia, hypothalamic inflammation, and type 2 diabetes mellitus (T2DM) [1, 2]. In animal models, a high-fat diet (HFD) causes obesity, hepatic steatosis, and insulin resistance [3, 4]. Particularly, dysregulation of energy homeostasis is caused by an imbalance between orexigenic and anorexigenic neuropeptides in the arcuate nucleus of the hypothalamus. HFD increases proopiomelanocortin (POMC) mRNA expression because food intake is lower in HFD-fed rodents than in chow-fed controls [5, 6].

Thiazolidinediones (TZDs), called glitazones, are prescribed to lower glucose in patients with T2DM. They also effectively delay T2DM onset when administered at during prediabetes [7]. This anti-diabetic effect of TZDs is mediated by increased glucose transporter 4 (GLUT4) mRNA expression and glucose uptake in skeletal muscle, liver, and adipose tissue via peroxisome proliferator-activated receptor γ (PPARγ), as well as by preserving pancreatic beta cell function [7–9]. Pioglitazone also has cardiovascular protective effects, as well as fat-lowering effects in central adipose tissue, such as the myocardium and liver [10]. However, clinical studies of TZDs have demonstrated an average 3–4 kg weight gain over the first 6 months. Glitazone-associated weight gain is attributed to various mechanisms, such as hyperphagia, increased fat mass, fluid retention, and improved glycemic control [11–13]. A PPARγ agonist increased food intake and weight in rodents, and overexpression and activation of the hypothalamic PPARγ isoform led to positive energy balance in rats [14]. This paradox of TZD-induced body fat gain but improved insulin resistance may be explained by differential effects on body fat distribution. TZDs reduce or do not increase visceral fat, but they induce subcutaneous fat deposition, causing body fat redistribution.

Lobeglitazone (Lobe) is a novel TZD that improves insulin resistance in T2DM [15]. It is a potent agonist of both PPARα and PPARγ, which enhances insulin sensitivity and decreases inflammation [16]. Lobe is currently prescribed for patients with T2DM in Korea [17]. It has clearly beneficial effects on insulin sensitivity; however, there have been no reports of positive effects on hepatic steatosis and weight gain in HFD-induced obese mice.

The purpose of the current study was to investigate whether Lobe alters insulin sensitivity, hepatic lipogenesis, and feeding activity in obese mice. We performed several metabolic studies and metabolomics to evaluate whether Lobe has positive effects on insulin resistance and hepatic steatosis in HFD-fed mice. We also evaluated leptin-mediated signaling transducer and activator of transcription 3 (STAT3) signaling in the hypothalamus to explore the effects of Lobe on weight gain in these mice.

## Materials and methods

### Animals and treatment

Male C57BL/6 mice (3 weeks old) were purchased from KOATECH (Pyeongtaek, South Korea) and maintained in the animal facility at Gyeongsang National University (GNU). Animal experiments were performed according to the National Institutes of Health Guidelines on the Use of Laboratory Animals. The Animal Care Committee for Animal Research of GNU approved the study protocol (GNU-150116-M0002). Mice were housed in a 12-h light/12-h dark cycle.

For the HFD-induced obesity model, mice were divided into three groups (n = 8 per group) at 3 weeks of age and fed a HFD (45 kcal% fat, 4.73 kcal/g, Research Diets, Inc., New Brunswick, NJ) for 15 weeks. Based on previous studies, Lobe 1 or 5 mg/kg/d (Chong Kun Dang Pharm, Seoul, South Korea) was administered orally for 9 weeks to two of the three HFD-fed groups, starting at 9 weeks of age [18]. Control normal diet (ND) mice (n = 8) were fed standard diet chow (2018S, 3.1 kcal/g, Harlan Laboratories, Inc., Indianapolis, IN). Lobe-only mice (n = 8) were fed a ND and received Lobe 5 mg/kg/d from 9 to 18 weeks of age. All rats were weighed every 3 weeks and just before sacrifice at age 18 weeks.

### Glucose tolerance test and insulin tolerance test

Mice were fasted overnight (16 h) before the glucose tolerance test (GTT) and 5 days after GTT, the insulin tolerance test (ITT) was performed at 2 p.m., as previously described [3]. In addition, we described the GTT and ITT test in detail as supplemental methods (S1 Methods).

### Measurement of body fat mass

The total body fat mass (fat and lean mass) was measured using magnetic resonance imaging (EchoMRI; Echo Medical Systems, Houston, USA).

### Measurement of serum metabolic parameters

All mice were anesthetized with Zoletil (5 mg/kg, Virbac Laboratories, Carros, France). Blood samples (n = 8 per group) were extracted transcardially through the apex of the left ventricle with a 1-mL syringe, and the blood was allowed to clot for 2 h at room temperature. After centrifugation, the serum was removed and stored at -80°C until analysis. Serum glucose, aspartate aminotransferase (AST), and alanine aminotransferase (ALT) levels were determined using enzymatic colorimetric assays from Green Cross Reference Laboratory (Yongin-si, South Korea). Serum insulin, leptin, and lipocalin-2 concentrations were measured using insulin (AKRIN-011T, Shibayagi, Gunma, Japan), leptin (AKRLP-011, Shibayagi), and lipocalin-2 (MLCN20, R&D systems, Minneapolis, MN, USA) mouse enzyme-linked immunosorbent assay (ELISA) kits, according to the manufacturers’ protocols. The homeostatic model assessment for insulin resistance (HOMA-IR) was calculated by multiplying fasting serum glucose (mg/dL) by fasting serum insulin (mU/ml) and dividing the total by 405.

### Tissue collection and histologic examination

Mice (n = 3 per group) were anesthetized with intramuscular Zoletil (Virbac Laboratories) and perfused with 4% paraformaldehyde in 0.1 M phosphate-buffered saline (PBS) for tissue analysis. After 6 h of fixation, mouse brains and livers were sequentially immersed in 15%, 20%, and 30% sucrose at 4°C until they sank. Brains and livers were then sliced into 40-μm and 5-μm sections, respectively. The livers and pancreases were processed for paraffin embedding and sliced into 5-μm sections. Deparaffinized liver sections were stained with hematoxylin and eosin (H&E). To determine hepatic lipid accumulation, frozen liver sections were stained with Nile Red (Sigma) for 10 min. Sections were visualized under a BX51 light microscope (Olympus, Tokyo, Japan). Digital images were captured and saved.

### Immunofluorescence

Free-floating brain sections and deparaffinized pancreas sections were placed in 0.3% H_2_O_2_ solution for 10 min. After washing, sections were treated with diluted blocking serum for 20 min. Slides were incubated with primary antibody (S1 Table) overnight at 4°C in a humidified chamber. After washing three times with 0.1 M PBS, sections were incubated with Alexa Fluor 488- and 594-conjugated donkey secondary antibodies (Invitrogen, Carlsbad, USA). A BX51-DSU microscope (Olympus) captured fluorescent images. In addition, we described immunohistochemistry for GLUT4 and PPARγ in detail as supplemental methods (S1 Methods).

### Western blot analysis

Western blot analyses were performed using standard methods. For protein extraction, frozen livers and hypothalamuses (n = 5 per group) were homogenized in lysis buffer. Proteins were immunoblotted with primary antibodies (S1 Table). Membranes were visualized using an enhanced chemiluminescence substrate (Pierce). To normalize protein levels, p84 or β-actin served as internal controls. Band densitometry was performed using the Multi-Gauge V 3.0 image analysis program (Fujifilm, Tokyo, Japan).

### Metabolomic analysis

Metabolite profiles of serum and liver tissues (n = 7 per group) were analyzed using ultra-performance liquid chromatography-quadrupole-time-of-flight (UPLC-Q-TOF) mass spectrometry (MS) (Waters, Milford, MA, USA). Liver and serum metabolites were extracted as previously described [19]. All MS data, including retention time, *m/z*, and ion intensity, were obtained using MarkerLynx software (Waters). Metabolite peaks were obtained using a peak width at 5% height of 1 s, noise elimination of 6, and intensity threshold of 10,000. Data were aligned with a 0.05 Da mass tolerance and retention time window of 0.2 s. All mass spectra were normalized to an internal standard. The metabolites were identified using human metabolome databases (www.hmdb.ca), the METLIN database (metlin.scripps.edu), ChemSpider (www.chemspider.com), literature references, and authentic standards.

### Statistical analysis

Differences between ND mice, HFD-fed mice receiving Lobe (1 mg/kg/d or 5mg/kg/d), HFD-fed mice not receiving Lobe, and Lobe-only mice were determined by one-way analysis of variance (ANOVA), followed by Tukey’s post hoc analysis. Repeated measures analysis of variance was used for food intake and body weight. Values are expressed as the mean ± standard error of the mean. *P* values <0.05 were considered statistically significant.

For metabolomics analysis, MS data sets underwent multivariate statistical analysis using SIMCA-P^+^ version 12.0.1 (Umetrics, Umeå, Sweden). Differences among sample groups were visualized by partial least squares-discriminant analysis (PLS-DA). PLS-DA models were evaluated by the goodness of fit (R2X and R2Y) and predictive ability (Q2Y), and validated by a 7-fold cross validation with a permutation test (n = 200). Metabolite intensity differences were analyzed by one-way ANOVA, with Duncan’s post hoc test (*p<*0.05), using SPSS 17.0 (SPSS Inc., Chicago, IL). Identified metabolites with significant differences (*p*<0.05) were visualized in a heat map drawn by R with ggplot2 representing the z-score transformed data of serum and liver metabolites. Pearson’s correlations between identified metabolites and serum and hepatic parameters were analyzed visualized by heat map.

## Results

### Lobeglitazone increased body weight and food intake in HFD-fed mice

**Fig 1:**
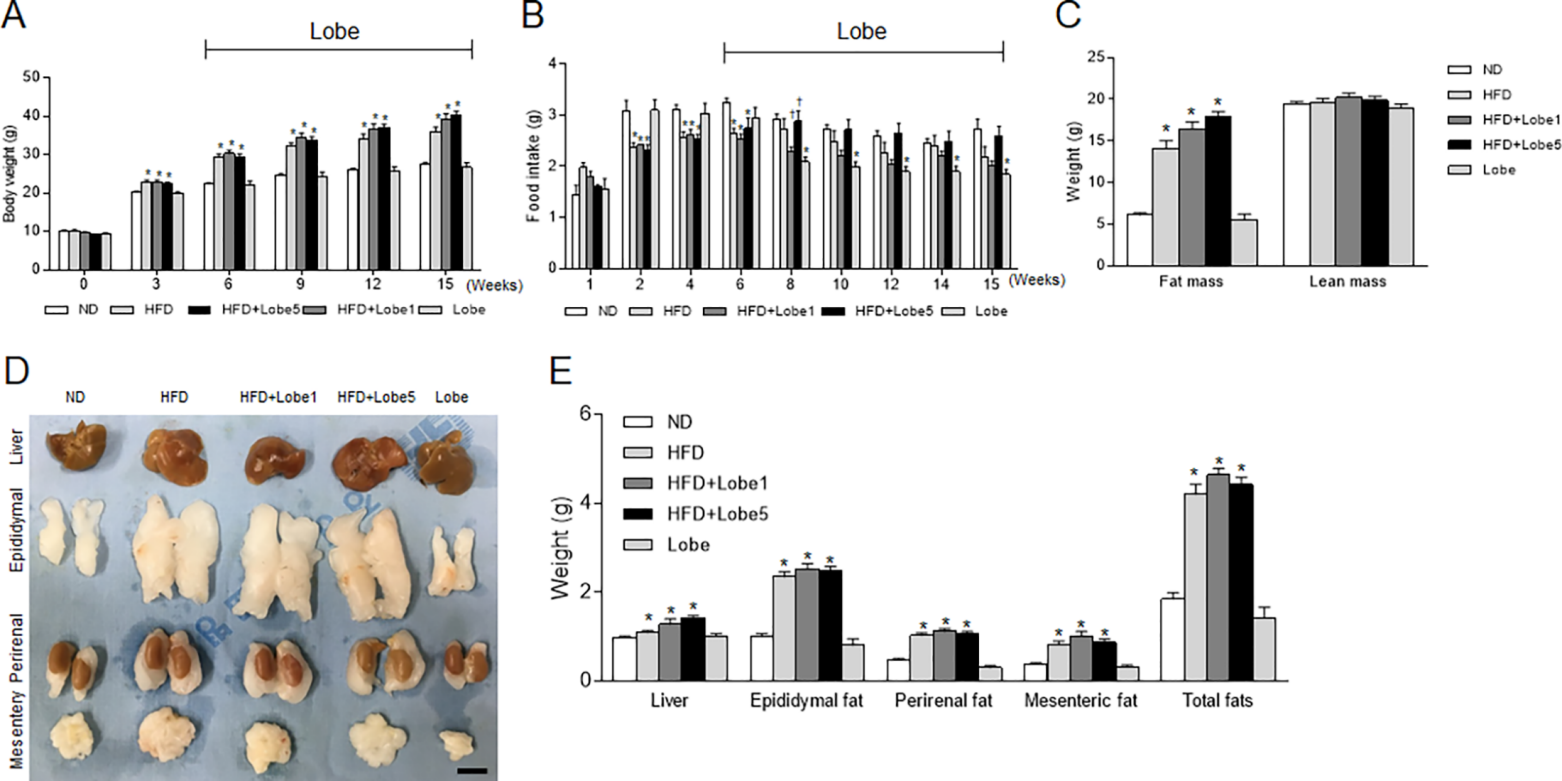
Effect of lobeglitazone (Lobe) on body weight and food intake in high-fat diet (HFD)-fed mice. Mice were fed Lobe (1 or 5 mg/kg/d) for 9 weeks (n = 8 per group). (A) Body weight, (B) food intake, (C) fat and lean mass, (D) representative photographs of intraabdominal organs, and (E) organ weights for each group at the time of sacrifice (18 weeks of age). Data are presented as mean ± SEM. *P <0.05 vs. normal diet (ND)-fed mice; †*P* <0.05 vs. HFD-fed mice. Scale bar = 1 cm.

After 6 weeks of HFD, Lobe 1 or 5 mg/kg/d was administered for 9 weeks. Body weight was significantly increased in HFD-fed mice compared with ND-fed mice (Fig 1A) and slightly increased in Lobe-treated HFD-fed mice relative to untreated HFD-fed mice. Although Lobe 5 mg/kg/d significantly decreased food intake (g) in Lobe-only mice (Fig 1B), change in body weight was similar in ND-fed and Lobe-only mice. In particular, Lobe 1 mg/kg/d or 5 mg/kg/d decreased or increased food intake (g) in HFD-fed mice 2 weeks after Lobe treatment, respectively. In particular, the reduction of food intake in Lobe-only mice did not affect weight change compared to ND-fed mice. Consistent with the body weight changes, food intake (kcal) was slight increase in Lobe 5 mg/kg/d-treated HFD-fed mice compared with untreated HFD-fed mice (Fig 1B and S1A Fig).

To determine the effects of Lobe treatment on weight gain in HFD-fed mice, we weighed the liver and fat mass including the epididymal fat pads, perirenal, and mesenteric fats (Fig 1C and 1E). Consistent with body weight gain, fat mass was a small rise in Lobe-treated HFD-fed mice compared to that in untreated HFD-fed mice (Fig 1C). Lobe did not reduce liver or intraabdominal fat weight in HFD-fed mice (Fig 1D and 1E).

### Lobeglitazone improved insulin sensitivity in HFD-fed mice

**Fig 2:**
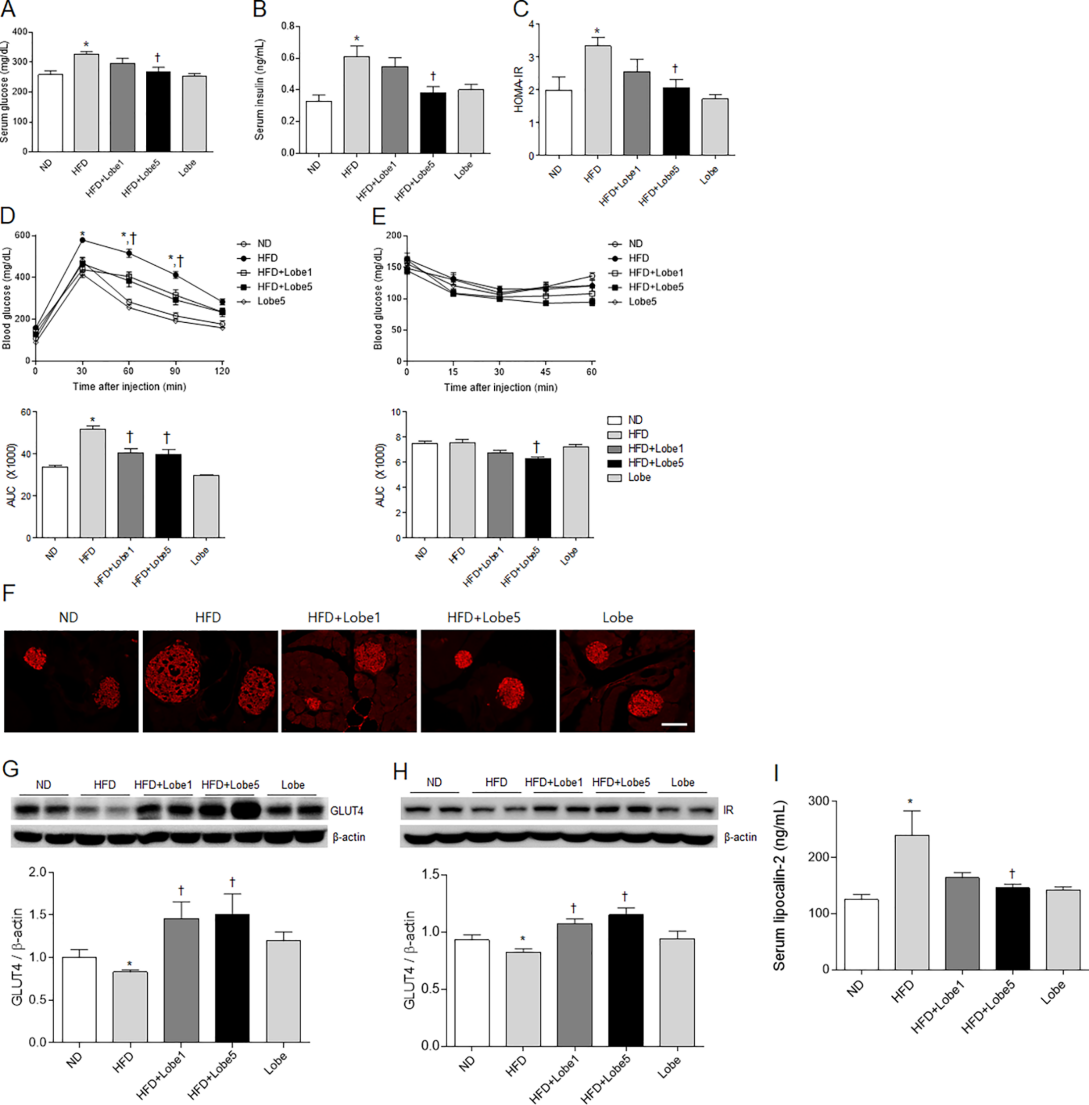
Effect of lobeglitazone (Lobe) on insulin resistance in high-fat diet (HFD)-fed mice. (A) Serum glucose, (B) serum insulin, (C) homeostatic model assessment of insulin resistance (HOMA-IR), (D) glucose tolerance test, and (E) insulin tolerance test in normal diet (ND), non-treated HFD, Lobe-treated (1 or 5 mg/kg/d) HFD, and Lobe-only mice. (F) Representative microphotographs of immunostained insulin in pancreatic sections from each group. Scale bar = 100 μm. Western blot for (G) glucose transporter 4 (GLUT4) and (H) insulin receptor (IR) expression in the liver for each group. Quantification of GLUT4 and IR expression from western blot analysis. Densitometry values are normalized to β-actin and represented as arbitrary units. (I) Serum lipocalin-2 concentrations obtained by enzyme-linked immunosorbent assay. Data are presented as mean ± SEM. *P <0.05 vs. ND-fed mice; †*P* <0.05 vs. HFD-fed mice. AUC, area under the curve.

Fasting blood glucose levels were significantly higher in HFD-fed mice than in ND-fed mice at 6 weeks after HFD (S1B Fig). After 9 weeks of Lobe 5 mg/kg/d, blood glucose levels were significantly decreased in HFD-fed mice. The effects of Lobe on serum glucose, insulin, and HOMA-IR levels in HFD-fed mice were examined (Fig 2A–2C). Hyperglycemia and hyperinsulinemia in HFD-fed mice were significantly reversed by only Lobe 5 mg/kg/d.

To explore the role of Lobe on insulin resistance in HFD-fed mice, GTT and ITT were performed (Fig 2D and 2E). Glucose tolerance in HFD-fed mice was significantly improved by Lobe (Fig 2D). ITT-AUC, there was no significant difference between ND-fed mice and HFD-fed mice (Fig 2E). However, only high-dose Lobe significantly improved insulin sensitivity in HFD-fed mice. Immunohistochemistry showed that HFD-induced hyperplasia in insulin-positive β-cells was reduced by Lobe (Fig 2F). To investigate the effect of Lobe on hepatic GLUT4 and insulin receptor (IR) expression, we performed western blot analysis (Fig 2G and 2H). Hepatic GLUT4 and IR expression was reduced in HFD-fed mice compared to ND-fed mice. After Lobe treatment, hepatic GLUT4 and IR levels increased significantly in HFD-fed mice. In addition, to test whether GLUT4 express in the liver sections, we performed immunohistochemistry. Immunohistochemical staining showed that Lobe increases the reduction of GLUT4-positive hepatocytes in the liver of HFD-fed mice (S2A and S2B Fig).

Serum lipocalin-2 is closely associated with obesity and T2DM in humans [20]. To investigate the effect of Lobe on serum lipocalin-2 in HFD-fed mice, we performed ELISA (Fig 2I). Lobe 5 mg/kg/d significantly reduced HFD-induced serum lipocalin-2 levels.

### Lobeglitazone promoted hepatic steatosis and lipogenesis in HFD-fed mice

**Fig 3:**
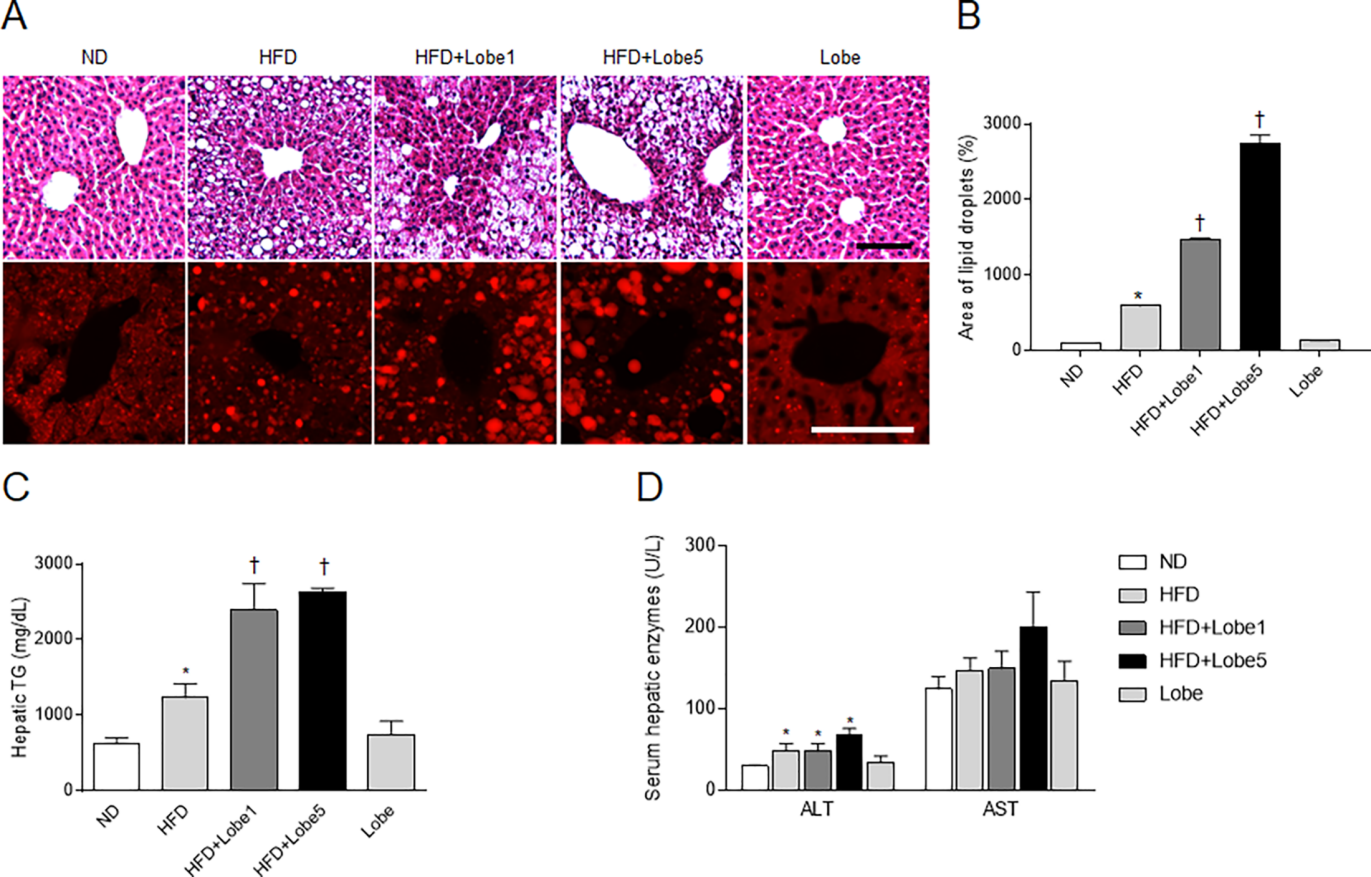
Effect of lobeglitazone (Lobe) on hepatic steatosis in high-fat diet (HFD)-fed mice. (A) Hematoxylin and eosin and Nile Red staining of hepatic lipid accumulation, (B) percentage of lipid droplets in Nile Red-stained sections, (C) hepatic triglyceride (TG) concentration, and (D) serum hepatic enzymes in normal diet (ND), non-treated HFD, Lobe-treated HFD (1 or 5 mg/kg/d), and Lobe-only mice. *P <0.05 vs. ND-fed mice; †*P* <0.05 vs. HFD-fed mice.

Hepatic steatosis is characterized by lipid droplet accumulation within hepatocytes and increased lipogenesis [21]. To assess the effects of Lobe on hepatic steatosis, we performed H&E and Nile Red staining (Fig 3A). Histology revealed hepatocytes distended with lipid droplets in HFD-fed mice. Unexpectedly, Lobe increased lipid droplets in the livers of HFD-fed mice (Fig 3B). Consistent with quantitative histomorphometry, hepatic triglyceride (TG) and serum liver enzyme levels in HFD-fed mice were not significantly inhibited by Lobe (Fig 3C and 3D).

**Fig 4:**
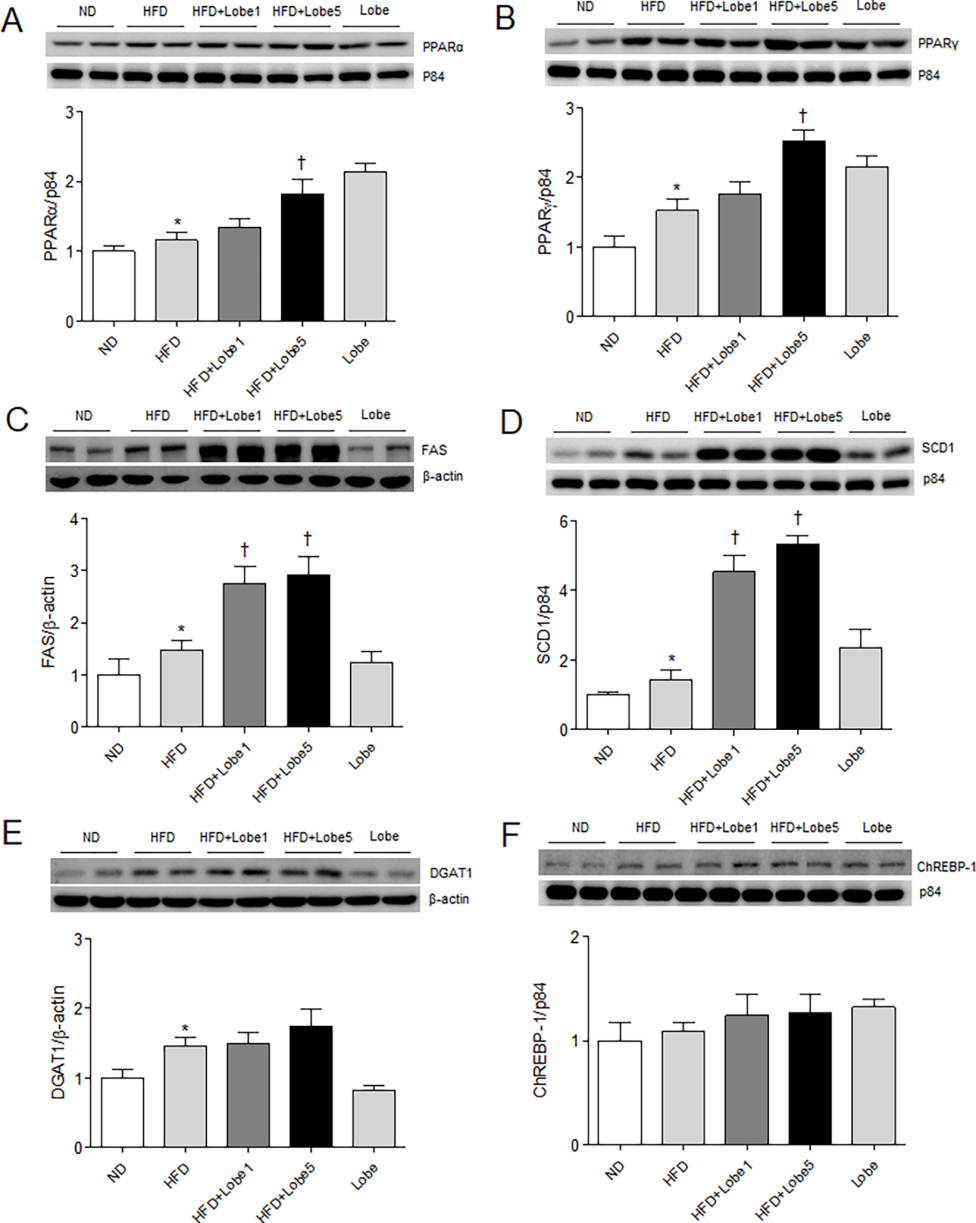
Effect of lobeglitazone (Lobe) on hepatic lipogenesis in high-fat diet (HFD)-fed mice. Western blots and quantification of hepatic (A) peroxisome proliferator-activated receptor (PPAR) α, (B) PPARγ, (C) fatty acid synthase (FAS), (D) stearoyl-CoA desaturase (SCD) 1, (E) diglyceride acyltransferase (DGAT) 1, and (F) carbohydrate response element binding protein (ChREBP)-1 expression in normal diet (ND), non-treated HFD, Lobe-treated HFD (1 or 5 mg/kg/d), and Lobe-only mice. Densitometry values for each protein are normalized to β-actin or p84. Data are presented as mean ± SEM. *P <0.05 vs. ND-fed mice; †*P* <0.05 vs. HFD-fed mice.

Synthesis of hepatic TG is regulated by key metabolic enzymes [22]. We evaluated the effects of Lobe on hepatic PPARα and PPARγ expression in HFD-fed mice (Fig 4A and 4B). Lobe, an agonist of PPARα and PPARγ, increased expression of both PPARs in HFD-fed mice. To evaluate whether PPARγ express in the liver sections, we performed immunohistochemistry. PPARγ-positive hepatocytes were stained more intensely for PPARγ in HFD-Lobe5-treated mice than those in HFD-fed mice (S2A and S2C Fig). Western blots showed increased lipogenesis proteins (fatty acid synthase [FAS], stearoyl-CoA desaturase [SCD] 1, and diglyceride acyltransferase [DGAT] 1) in the livers of HFD-fed mice compared to ND-fed mice (Fig 4C–4E). However, Lobe did not reduce these HFD-induced changes; conversely, Lobe increased these proteins in a dose-dependent manner. Hepatic carbohydrate response element binding protein (ChREBP)-1 expression did not differ between groups (Fig 4F).

### Lobeglitazone altered hepatic and serum metabolites in HFD-fed mice

**Fig 5:**
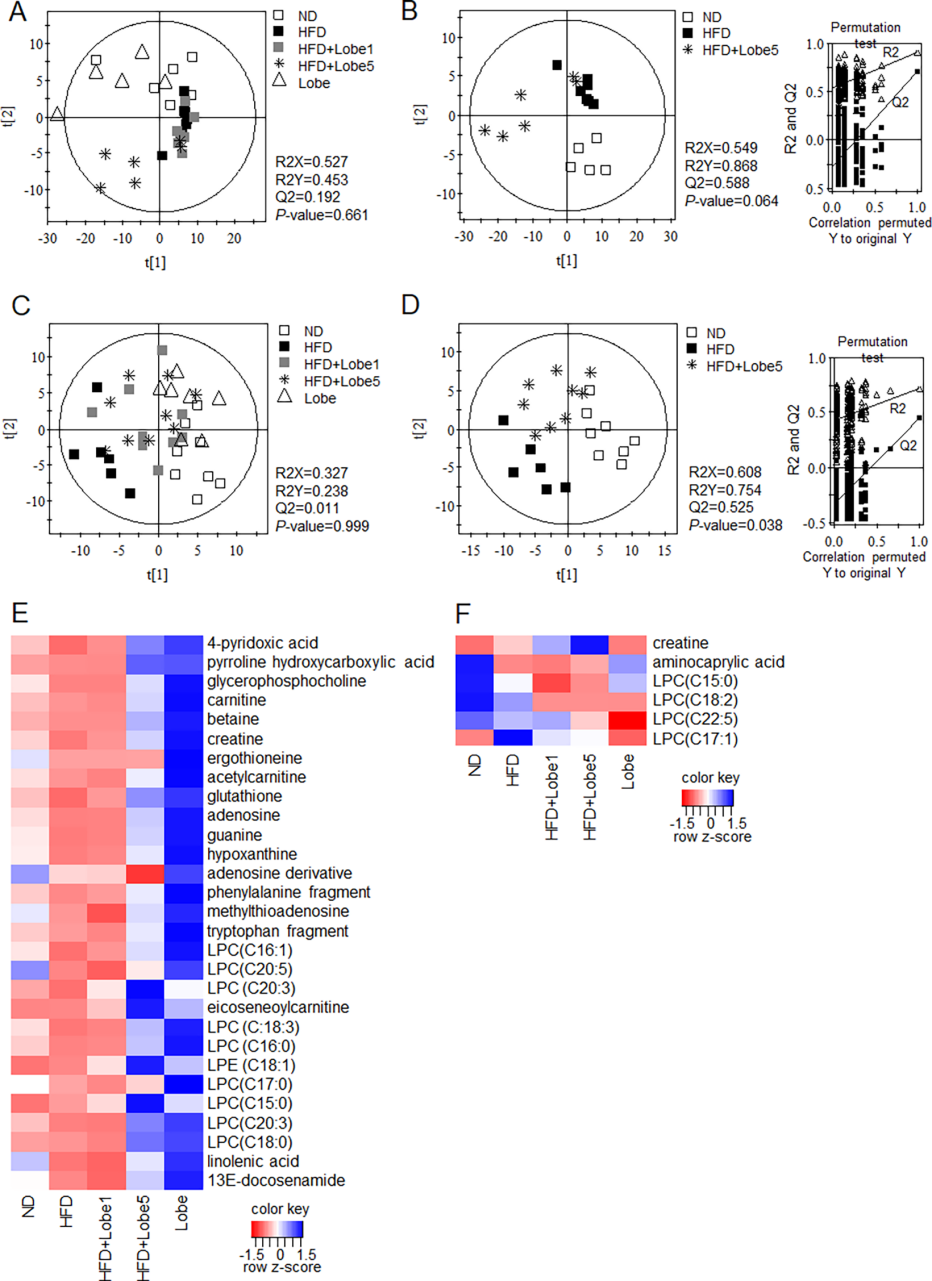
**Partial least squares-discriminant analysis (PLS-DA) scores scatter plots of liver (A and B) and serum (C and D) metabolites analyzed using ultra-performance liquid chromatography-quadrupole-time-of-flight mass spectrometry and (UPLC-Q-TOF MS) and heat maps of identified hepatic (E) and serum (F) metabolites.** Differences among all sample groups (A; liver, C; blood) and sample groups of ND, HFD, and HFD+ lobeglitazone 5 mg (B; liver, D; blood) were visualized respectively by PLS-DA scores scatter plots. The quality of the PLS-DA models were evaluated by the goodness of fit (R2X and R2Y) and predictive ability (Q2Y), and validated by a 7-fold cross validation with a permutation test (n = 200). All sample groups were visualized by Heat maps of identified hepatic (E) and serum (F) metabolites obtained using UPLC-Q-TOF MS. The heat maps were drawn using R with ggplot2, and the red-blue colors represent the z-score transformed raw data of serum and hepatic metabolites with significant differences among groups. Red and blue indicate decreases and increases in metabolite levels, respectively. HFD, high-fat diet; Lobe, lobeglitazone; LPC, lysophosphatidylcholine; ND, normal diet.

As Lobe did not reverse HFD-induced hepatic lipogenesis and increased TG, we performed UPLC-Q-TOF MS to determine whether Lobe affects serum and hepatic metabolites of HFD-fed mice. The MS data were analyzed statistically by partial least squares discriminant analysis (PLS-DA) (Fig 5). Low-quality parameters (R2X, R2Y, and Q2) of the PLS-DA models for all groups (ND, HFD, HFD+Lobe 1 mg/kg/d, HFD+Lobe 5 mg/kg/d, and Lobe-only), as well as the *P*-values (0.661 and 0.999 for liver and serum, respectively) (Fig 5A and 5C), indicated that both models for all groups were not acceptable for further analysis. However, the model quality parameters (R2X = 0.549, R2Y = 0.868, and Q2 = 0.588 for liver; R2X = 0.608, R2Y = 0.754, and Q2 = 0.525 for serum) (Fig 5B and 5D), *P*-values (0.064 and 0.038 for liver and serum, respectively), and values obtained from cross-validation by a permutation test (R2 and Q2 intercepts) indicated that the PLS-DA plots for three groups (ND, HFD, and HFD+Lobe 5 mg/kg/d) were statistically acceptable. Based on these parameters, the three groups were clearly separated from each other on the first 2-component PLS-DA score plots (Fig 5B and 5D for liver and serum, respectively). Hepatic and serum metabolites contributing to the separation were identified (S2 Table). Among identified metabolites, lysophosphatidylcholine (LPC; C18:2) and aminocaprylic acid from serum and 13E-docosenamide, ergothioneine, adenosine derivative, LPCs (C15:0), LPC (C20:5), and lysophosphatidylethanolamine (LPE; C18:1) from liver, with variable importance in the projection values >1.00 and *P*-values <0.05, were highly relevant and contributed to the discrimination among ND, HFD, and HFD+Lobe 5 mg on the PLS-DA score plot. The identified serum and liver metabolite profiles for the ND, HFD, HFD+Lobe 1 mg, HFD+Lobe 5 mg, and Lobe-only groups were compared using heat maps (Fig 5E and 5F) and box plots (S3 Fig). Levels of most hepatic and serum metabolites altered by HFD were moderated by Lobe 5 mg/kg/d (not Lobe 1 mg/kg/d), whereas Lobe treatment alone increased the levels of all identified metabolites (Fig 5E and 5F). In the liver, levels of LPCs (C16:0, C16:1, C17:0, C18:0, C18:3, C20:3, C20:5), LPE (C18:1), glutathione, docosenamide, adenosine, glycerophosphocholine, hypoxanthine, phenylalanine, creatine, eicoseneoylcarnitine, methylthioadenosine, 4-pyridoxic acid, guanine, tryptophan, and linolenic acid were significantly decreased by HFD. These hepatic metabolites were significantly reversed by Lobe 5 mg/kg/d (S3A Fig). Lobe did not reverse decreased hepatic ergothioneine in HFD-fed mice. In serum, aminocaprylic acid was significantly decreased by HFD, whereas creatine, LPC (C15:0), and LPC (C17:1) were increased. However, only LPC (C18:2) was decreased by Lobe 5 mg/kg/d (S3B Fig). Additionally, there were positive correlations between identified metabolites and metabolic parameters in HFD-fed mice (S4 Fig). Among hepatic metabolites, LPE (C18:1) was closely correlated with hepatic TG and serum ALT levels. Serum LPC (C17:1) levels were positively correlated with serum insulin and lipocalin-2 levels.

### Lobeglitazone increased phosphorylation of STAT3 in the hypothalamus in HFD-fed mice

**Fig 6:**
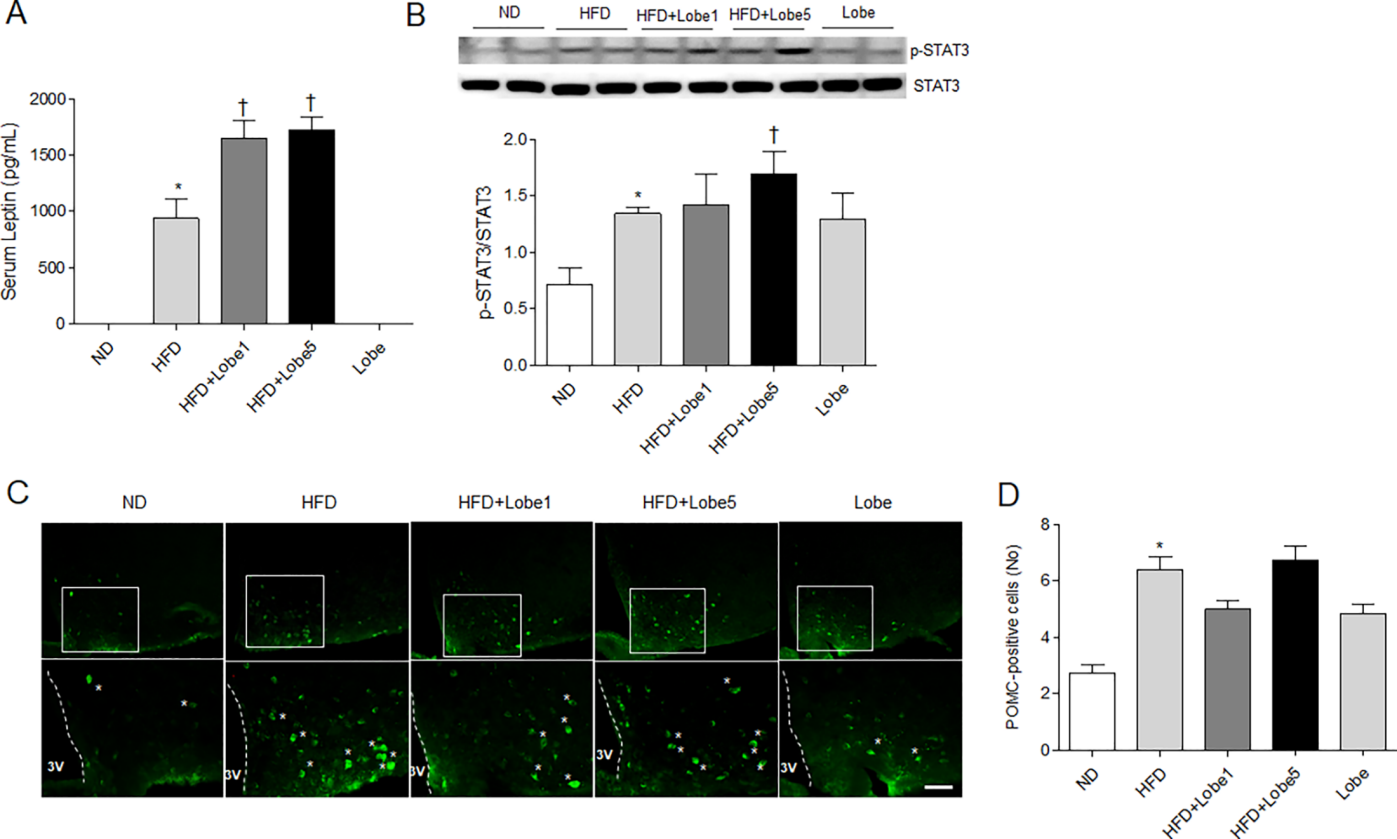
Effect of lobeglitazone (Lobe) on serum leptin and hypothalamic signaling transducer and activator of transcription 3 (STAT3) and proopiomelanocortin (POMC) 2 expression in HFD-fed mice. (A) Serum leptin concentrations and (B) western blot and quantification of p-STAT3 and total STAT3 in the hypothalamuses from each group. Densitometry values for each protein are normalized to total STAT3. (C) Representative microphotographs of immunostained POMC-positive cells in the arcuate nucleus of hypothalamuses from each group. Asterisks indicate POMC-positive neurons. Scale bar = 50 μm. (D) The number of POMC-positive cells. Data are presented as mean ± SEM. *P <0.05 vs. ND-fed mice; †*P* <0.05 vs. HFD-fed mice. HFD, high-fat diet; Lobe, lobeglitazone; ND, normal diet.

To determine the effects of Lobe administration on food intake in HFD-fed mice, we measured serum leptin levels using ELISA (Fig 6A). Compared to ND-fed and Lobe-fed mice, serum leptin levels were significantly increased in HFD-fed mice. Unexpectedly, both Lobe doses significantly induced serum leptin levels compared to HFD-fed mice. Thus, to determine whether Lobe affects leptin-mediated STAT3 signaling, we evaluated hypothalamic p-STAT3 using western blot analysis (Fig 6B). Consistent with leptin concentrations, Lobe 5 mg/kg/d upregulated p-STAT3 in the hypothalamus of HFD-treated mice. To evaluate the effects of Lobe on hypothalamic POMC-positive neurons in HFD-fed mice, we performed immunohistochemistry (Fig 6C and 6D). There were many POMC-positive neurons in the hypothalamus of HFD-fed mice (with or without Lobe) compared to ND-fed mice (Fig 6D). Lobe treatment did not affect the number of these neurons in HFD-fed mice. Western blot analysis demonstrated no change in hypothalamic leptin receptor, POMC, and PPARγ expression in HFD-fed mice with or without Lobe treatment (data not shown).

## Discussion

Lobe is a novel TZD with antidiabetic effects that are not completely understood. A recent study reported that Lobe improved non-alcoholic fatty liver disease (NAFLD) in patients with T2DM [15]. The current study revealed that although Lobe improved insulin resistance in HFD-fed mice, HFD-induced hepatic steatosis and weight gain were not reversed by Lobe. Thus, we suggest that the dose and administration duration of Lobe be considered with caution in diabetic patients with hepatic steatosis.

Several randomized trials in humans reported the hypoglycemic efficacy of Lobe for T2DM [17, 23–25]. As monotherapy, Lobe significantly decreased hemoglobin A1c and improved fasting plasma glucose, insulin, and HOMA-IR [23, 25]. Lobe plus metformin showed similar beneficial effects on glycemic control and insulin resistance in patients with T2DM [24]. Consistent with these results, we found significantly improved fasting glucose, serum insulin, and HOMA-IR in HFD-fed mice treated with Lobe (5 mg/kg/d), compared to untreated HFD-fed mice. Accompanying these hypoglycemic changes, Lobe reduced insulin-positive pancreatic β-cell hyperplasia and reversed reduction of GLUT4 and IR expression in the liver of HFD-fed mice. GLUT4 plays an important role in maintaining glucose homeostasis [26], with higher GLUT4 expression preventing insulin resistance. Our results showed that insulin sensitivity and glucose utilization improved significantly in Lobe-treated HFD-fed mice through increased GLUT4 expression. The hypoglycemic action of Lobe appears to be as effective in HFD-fed mice as in T2DM patients.

The most common adverse effect of TZDs in patients is weight gain [27–30]. Some reports suggest that weight gain is due to fluid retention [31, 32]. Here, we found a slight Lobe-induced increase in total body water using EchoMRI (data not shown). Furthermore, Lobe-treated HFD-fed mice exhibited a slightly greater increase in body weight and food intake (kcal) than untreated HFD-fed mice. Body fat mass, including mesenteric, perirenal, and epididymal fat pads, in Lobe-treated HFD-fed mice were higher than in untreated HFD-fed mice. As PPARγ agonists, TZDs cause adipogenesis and promote preadipocyte to adipocyte differentiation [33, 34].

Our HFD-fed mice exhibited more hepatocyte lipid droplets and higher hepatic TG and serum hepatic enzyme levels than ND-fed mice. Furthermore, Lobe-treated HFD-fed mice showed more lipid accumulation and higher hepatic TG and enzymes than untreated HFD-fed mice. Serum leptin was higher in HFD-fed mice than in ND-fed mice. In Lobe-treated HFD-fed mice, changes in this adipokine were more prominent. Lipocalin-2 plays a critical role in regulating energy metabolism, glucose and lipid homeostasis, and insulin resistance [35,36]. Jin et al. demonstrated that lipocalin-2 is a selective modulator of PPARγ activation in lipid homeostasis [35]. Serum lipocalin-2 is closely associated with obesity and T2DM in humans [20]. In the present study, HFD-induced upregulation of serum lipocalin-2 was significantly reduced by Lobe 5 mg/kg/d, suggesting that serum lipocalin-2 levels are associated with improved insulin sensitivity during Lobe treatment.

Hepatic lipogenesis is catalyzed by enzymes such as FAS, SCD, and DGAT, which are regulated by ChREBPs [21, 37]. In the current study, these lipogenesis enzymes tended to increase with Lobe treatment in HFD-mice. We also confirmed elevated hepatic PPARα and PPARγ expression with Lobe in HFD-fed mice. While HFD causes hepatic lipogenesis, Lobe may aggravate hepatic lipogenesis because of enhanced adipogenesis via Lobe-induced PPARγ activation.

To further determine whether Lobe affects serum and liver metabolites in HFD-fed mice, serum and hepatic metabolite profiles were examined by UPLC-Q-TOF MS analysis. Among these metabolites, decreased ergothioneine in HFD-fed mice was not reversed by either dose of Lobe. As ergothioneine exhibited antioxidant activity *in vitro* [38], our finding suggests that hepatic steatosis is associated with lower antioxidant activity, which was not improved by Lobe. Glutathione, an important antioxidant, was also reduced by HFD, but Lobe 5 mg/kg/d increased hepatic glutathione in HFD-fed mice. Creatine, which is synthesized in the liver, is transported through the blood [39]. Compared with ND-fed mice, HFD-fed mice had a lower hepatic creatine but higher serum creatine. However, Lobe did not reverse the elevated serum creatine. These data suggest that creatine may be secreted into the blood by other organs in addition to the liver.

In addition to increased adipogenesis and fluid retention, TZDs may produce weight gain by hyperphagia [12, 13]. Although pioglitazone improved insulin resistance and hypothalamic leptin action in HFD-fed mice, it increased food intake via activation of the hypothalamic adiponectin receptor 1/AMP-activated protein kinase pathway [13]. In the present study, we also demonstrated increased weight gain and hepatic steatosis with Lobe despite reduced insulin resistance. Serum leptin levels are increased in patients with obesity and insulin resistance. Our findings showed that serum leptin, which circulates in the blood in proportion to body weight and fat mass, was increased in HFD-fed mice but did not decrease with Lobe.

Leptin-induced signal transduction via Janus kinase (JAK)-STAT signaling in the hypothalamus directly activates POMC neurons [40]. In line with this fact, inactivation of leptin receptor or dephosphorylation of STAT3 in POMC neurons reduces energy expenditure and increased food intake, thereby causing obesity in db/db mice [40, 41]. We hypothesize that HFD-induced leptin secretion (with or without Lobe treatment) might be both a consequence and a cause of obesity. We found increased hypothalamic p-STAT3 in parallel with the increase in serum leptin in HFD-fed mice. Furthermore, high-dose Lobe upregulated serum leptin levels (leptin insensitivity) and hypothalamic p-STAT3 expression in HFD-fed mice with weight gain. However, despite increased serum leptin concentration, low-dose Lobe decreased food intake compared to HFD-fed mice. In particular, although only high-dose Lobe treatment decreased food intake in ND-fed mice, there was no effect of hypophasia on serum leptin level. Thus, these data indicate that high-dose Lobe may directly affect hypothalamic STAT3 expression and increase food intake in HFD-fed mice, whereas low-dose Lobe decreases food intake and its effect on food intake appears to be inconsistent with serum leptin concentrations. We suggest that the dose and duration of Lobe treatment may be involved in mediating leptin effects on food intake in the hypothalamus.

In conclusion, Lobe treatment enhanced insulin sensitivity via GLUT4, but HFD-induced hepatic lipogenesis was not reversed by Lobe. Lobe affected regulation of food intake in the hypothalamus, thereby causing weight gain. Thus, careful consideration may be warranted before beginning monotherapy with Lobe for patients with T2DM and NAFLD.

## Supporting information

S1 Fig Effect of Lobe on food intake and fasting blood glucose in HFD-fed mice. Mice were fed Lobe (1 or 5 mg/kg/d) for 9 weeks (n = 8 per group). (A) Food intake (kcal) and (B) fasting blood glucose for each group during 15 weeks. Data are presented as mean ± SEM. *P <0.05 vs. normal diet (ND)-fed mice; †*P* < 0.05 vs. HFD-fed mice.
(TIF)

S2 Fig Effect of Lobe on hepatic GLUT4 and PPARγ expression in high-fat diet (HFD)-fed mice. (A) Representative micrographs of GLUT4 and PPARγ immunoreactivity in the liver sections. The intensity of GLUT4 (B) and PPARγ (C) immunoreactivity in the livers were measured and presented as the density. Data are presented as mean ± SEM. *P <0.05 vs. normal diet (ND)-fed mice; †*P* <0.05 vs. HFD-fed mice. Scale bar = 50μm.
(TIF)

S3 Fig Effect of Lobe on hepatic and serum metabolites in HFD-fed mice. Relative abundance of hepatic (A) and serum (B) metabolites analyzed using ultra-performance liquid chromatography-quadrupole-time-of-flight mass spectrometry in ND (N), HFD (H), HFD+Lobe 1mg/kg/d (H1), HFD+Lobe 5 mg/kg/d (H5), and Lobe-only (L) mice. HFD, high-fat diet; Lobe, lobeglitazone; LPC, lysophosphatidylcholine; LPE, lysophosphatidylethanolamine; ND, normal diet.
(TIF)

S4 Fig **Pearson’s correlations between metabolic parameters and (A) hepatic and (B) serum metabolites in HFD-fed mice.** Correlations were calculated using Excel and visualized using R with ggplot2. Red and blue colors indicate a negative and a positive correlation, respectively. ALT, alanine aminotransferase; LPC, lysophosphatidylcholine; LPE, lysophosphatidylethanolamine; TG, triglyceride.
(TIF)

S1 Table List of primary and secondary antibodies. (DOCX)

S2 Table Identification of blood and hepatic metabolites affected by HFD and Lobe treatment. (DOCX)

S1 Methods Additional detailed methods including immunohistochemistry. (DOCX)
