# Supplementary figures and images for: Effects of lobeglitazone on insulin resistance and hepatic steatosis in high-fat diet-fed mice
Source: PLoS One. 2018 Jul 6;13(7):e0200336. doi: 10.1371/journal.pone.0200336 (PMC6034891; doi:10.1371/journal.pone.0200336)

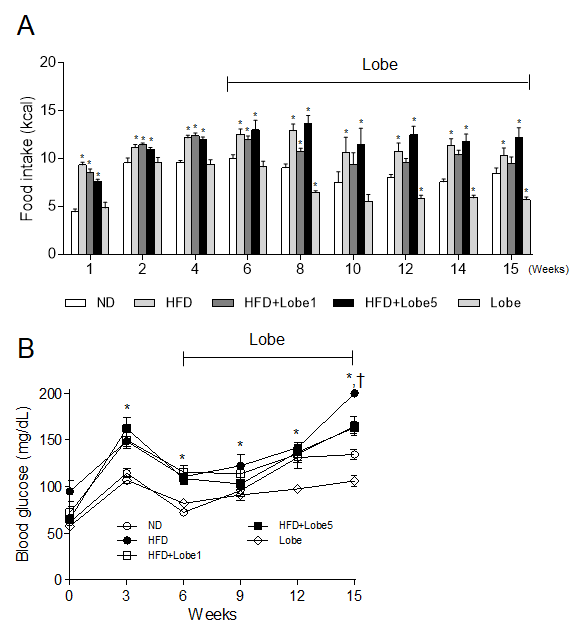

Supplement: S1 Fig — Mice were fed Lobe (1 or 5 mg/kg/d) for 9 weeks (n = 8 per group). (A) Food intake (kcal) and (B) fasting blood glucose for each group during 15 weeks. Data are presented as mean ± SEM. *P <0.05 vs. normal diet (ND)-fed mice; †P < 0.05 vs. HFD-fed mice. (TIF) [file pone.0200336.s001.tif]

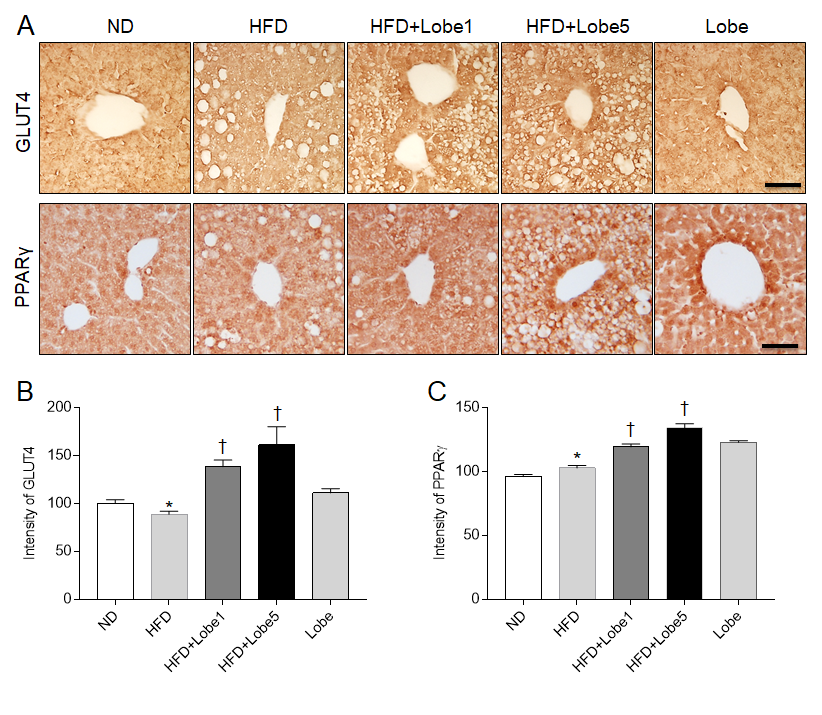

Supplement: S2 Fig — (A) Representative micrographs of GLUT4 and PPARγ immunoreactivity in the liver sections. The intensity of GLUT4 (B) and PPARγ (C) immunoreactivity in the livers were measured and presented as the density. Data are presented as mean ± SEM. *P <0.05 vs. normal diet (ND)-fed mice; †P <0.05 vs. HFD-fed mice. Scale bar = 50μm. (TIF) [file pone.0200336.s002.tif]

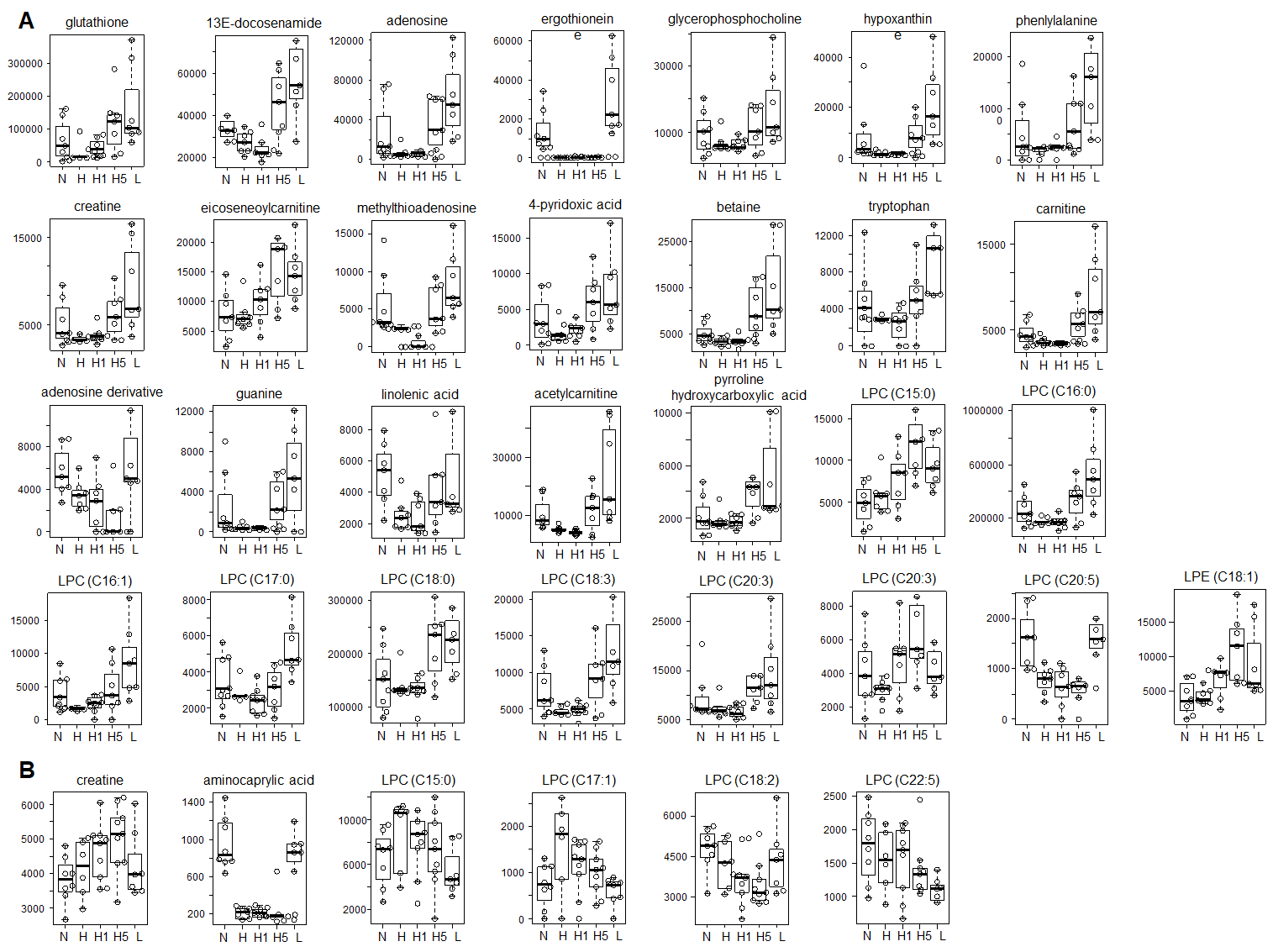

Supplement: S3 Fig — Relative abundance of hepatic (A) and serum (B) metabolites analyzed using ultra-performance liquid chromatography-quadrupole-time-of-flight mass spectrometry in ND (N), HFD (H), HFD+Lobe 1mg/kg/d (H1), HFD+Lobe 5 mg/kg/d (H5), and Lobe-only (L) mice. HFD, high-fat diet; Lobe, lobeglitazone; LPC, lysophosphatidylcholine; LPE, lysophosphatidylethanolamine; ND, normal diet. (TIF) [file pone.0200336.s003.tif]

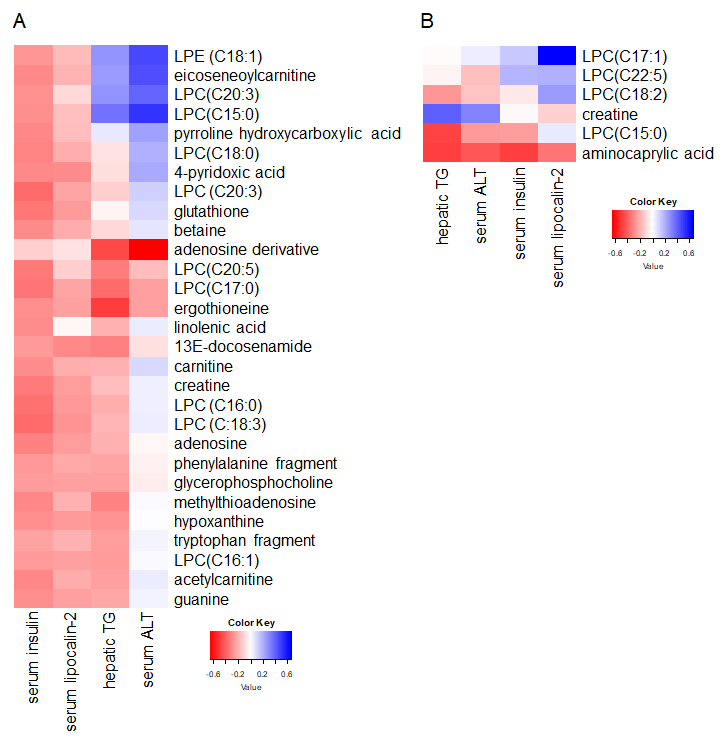

Supplement: S4 Fig — Pearson’s correlations between metabolic parameters and (A) hepatic and (B) serum metabolites in HFD-fed mice. Correlations were calculated using Excel and visualized using R with ggplot2. Red and blue colors indicate a negative and a positive correlation, respectively. ALT, alanine aminotransferase; LPC, lysophosphatidylcholine; LPE, lysophosphatidylethanolamine; TG, triglyceride. (TIF) [file pone.0200336.s004.tif]
