# Supplementary material for: Effects of lobeglitazone on insulin resistance and hepatic steatosis in high-fat diet-fed mice
Source: PLoS One. 2018 Jul 6;13(7):e0200336. doi: 10.1371/journal.pone.0200336 (PMC6034891; doi:10.1371/journal.pone.0200336)
Supplement: S1 Table — (DOCX) [file pone.0200336.s005.docx]

**S1 Table. List of primary and secondary antibodies**

| **Antibody** | **Company** | **Catalog No.** | **Dilution** | **Applications** | **Source** |
| --- | --- | --- | --- | --- | --- |
| Insulin | Abcam | Ab7842 | 1:100 | IF | Rabbit |
| IR | Millipore | MABS65 | 1:1000 | WB | Mouse |
| p-STAT3 | Cell signaling | #9131 | 1:1000 | WB | Rabbit |
| STAT3 | Cell signaling | #9132 | 1:1000 | WB | Rabbit |
| ChREBP1 | Novus | NB400-135 | 1:1000 | WB | Rabbit |
| DGAT1 | Santa Cruz | Sc-32861 | 1:1000 | WB | Rabbit |
| FAS | Cell signaling | #3189 | 1:1000 | WB | Rabbit |
| PPARα | Abcam | Ab8934 | 1:1000 | WB | Rabbit |
| PPARγ | Santa Cruz | Sc-7196 | 1:1000  1:200 | WB,  IHC | Rabbit |
| SCD1 | Cell signaling | #2438 | 1:1000 | WB | Rabbit |
| POMC | Phoenix | H-029-30 | 1:200 | IF | Rabbit |
| GLUT4 | Abcam | Ab654 | 1:1000  1:200 | WB,  IHC | Rabbit |
| p84 | Abcam | Ab487 | 1:1000 | WB | Mouse |
| β-actin | Sigma | A5441 | 1:3000 | WB | Mouse |
| IgG | Thermo | 31460 | 1:200 | IHC | Goat |

ChREBP1, carbohydrate response element binding protein 1; DGAT1, diglyceride acyltransferase 1; FAS, fatty acid synthase; GLUT4, glucose transporter 4; IF, immunofluorescence; IR, insulin receptor; POMC, proopiomelanocortin; PPAR, peroxisome proliferator-activated receptor; SCD1, stearoyl-CoA desaturase 1; WB, western blot
