# Supplementary material for: Effects of lobeglitazone on insulin resistance and hepatic steatosis in high-fat diet-fed mice
Source: PLoS One. 2018 Jul 6;13(7):e0200336. doi: 10.1371/journal.pone.0200336 (PMC6034891; doi:10.1371/journal.pone.0200336)
Supplement: S2 Table — (DOCX) [file pone.0200336.s006.docx]

**S2 Table.** **Identification of hepatic and serum metabolites affected by HFD and Lobe treatment**

|  | Compound | Exact mass | MS Fragments | *p*-value^a)^ | VIP^b)^ |
| --- | --- | --- | --- | --- | --- |
|  |  | (M+H) |  |  |  |
| **Liver** | glutathione | 308.0913 | 479, 162 | 0.032 | 0.713 |
|  | 13E-docosenamide | 338.3418 | 321, 303 | 0.044 | 1.142 |
|  | adenosine | 268.1042 | 136 | 0.040 | 0.798 |
|  | ergothioneine | 230.0959 | 127 | 4.638e-07 | 1.603 |
|  | glycerophosphocholine | 258.1103 | 240, 88 | 0.360 | 0.880 |
|  | hypoxanthine | 137.0459 | 119, 110, 94, 82 | 0.048 | 0.859 |
|  | phenylalanine fragment | 120.0808 | 120 | 0.051 | 0.752 |
|  | creatine | 132.0768 | 90 | 0.031 | 0.758 |
|  | eicoseneoylcarnitine | 454.2927 | 85 | 0.008 | 0.876 |
|  | methylthioadenosine | 298.0969 | 136 | 0.078 | 0.928 |
|  | 4-pyridoxic acid | 184.0735 | 166, 148 | 0.020 | 0.712 |
|  | betaine | 118.0862 | 102, 70, 52 | 0.014 | 0.829 |
|  | tryptophan fragment | 188.0707 | 146, 118 | 0.323 | 0.907 |
|  | carnitine | 162.1125 | 103, 85, 60 | 0.030 | 0.725 |
|  | adenosine derivative | 136.0619 | 136 | 0.052 | 1.323 |
|  | guanine | 152.0568 | 135, 110 | 0.032 | 0.804 |
|  | pyrroline hydroxycarboxylic acid | 130.0500 | 112 | 0.005 | 0.801 |
|  | acetylcarnitine | 204.1231 | 85 | 0.096 | 0.891 |
|  | linolenic acid | 279.2320 | 250, 211 | 0.096 | 1.178 |
|  | LPC(C15:0) | 482.3242 | 184, 104 | 0.002 | 1.041 |
|  | LPC (C16:0) | 496.3396 | 184, 104 | 0.042 | 0.738 |
|  | LPC(C16:1) | 494.3237 | 184, 104 | 0.247 | 0.747 |
|  | LPC(C17:0) | 510.3555 | 184, 104 | 0.989 | 1.246 |
|  | LPC (C18:0) | 524.3714 | 184, 104 | 0.017 | 0.803 |
|  | LPC (C18:3) | 518.3215 | 184, 104 | 0.043 | 0.789 |
|  | LPC (C20:3) | 546.3553 | 184, 104 | 0.014 | 0.858 |
|  | LPC(C20:5) | 542.3218 | 184, 104 | 0.003 | 1.210 |
|  | LPE (C18:1) | 480.3084 | 426, 337, 126 | 0.001 | 1.037 |
| **Serum** | aminocaprylic acid | 160.1334 | 114 | 5.44e-7 | 1.132 |
|  | creatine | 132.0768 | 90 | 0.048 | 0.738 |
|  | LPC(C15:0) | 482.323 | 184, 104 | 0.009 | 0.864 |
|  | LPC(C17:1) | 508.3756 | 184, 104 | 0.025 | 0.726 |
|  | LPC(C18:2) | 520.3401 | 184, 104 | 0.052 | 5.964 |
|  | LPC(C22:5) | 570.3543 | 184, 104 | 0.0203 | 0.404 |

a) *P*-values were analyzed by analysis of variance.

b) A variable importance in the projection (VIP) value above 1.00 represents high relevance for explaining the differences among sample groups.

LPC, lysophosphatidylcholine; LPE, lysophosphatidylethanolamine; MS, mass spectrometry
