# Supplementary material for: Effects of lobeglitazone on insulin resistance and hepatic steatosis in high-fat diet-fed mice
Source: PLoS One. 2018 Jul 6;13(7):e0200336. doi: 10.1371/journal.pone.0200336 (PMC6034891; doi:10.1371/journal.pone.0200336)
Supplement: S1 Methods — (DOCX) [file pone.0200336.s007.docx]

**Additional methods**

**Glucose tolerance test (GTT)**

Mice were fasted overnight (16 hrs) before GTT. D-glucose (2 g/kg, Sigma-Aldrich, St. Louis, MO, USA) was injected intraperitoneally and blood samples were taken before and 30, 60, 90, and 120 min after the injection of glucose. Blood glucose was measured using an Accu-Chek glucometer (Roche Diagnostics GmbH, Mannheim, Germany).

**Insulin tolerance test (ITT)**

The ITT was performed on mice around 2 pm. Mice were injected with insulin (0.75 U/kg, Humulin-R, Eli Lilly, Indianapolis, IN, USA) in 0.1 ml 0.9% normal saline. A drop of blood was taken from the cut tail vein before and 15, 30, 45, and 60 min after the injection of insulin for the determination of blood glucose with a glucometer (Accu-Chek).

**Immunohistochemistry**

Deparaffinized liver sections were placed in 0.3% H_2_O_2_ solution for 10 min. After washing, sections were treated with diluted blocking serum for 20 min. Slides were incubated with GLUT4 and PPARγ antibody (S1 Table) overnight at 4°C in a humidified chamber. After washing three times with 0.1 M PBS, sections were incubated with secondary antibodies (S1 Table) for 2 hours at room temperature. Image of the stained sections were captured using a BX51 light microscope (Olympus). Immunohistochemical intensity data for GLUT4 and PPARγ were obtained from selected images using i-Solution (IMT i-Solution Inc., Vancouver, BC, Canada). Three fields (200 × 200 µm^2^) were randomly selected on each section from two continuous sections (n = 3 per group). Intensity measurements are represented as the percentage of the mean number of pixels versus the corresponding value at which the pixel of the respective intensity was present.
